# Effect of Sodium Alginate Concentration on the Physicochemical, Structural, Functional Attributes, and Consumer Acceptability of Gel Beads Encapsulating Tangerine Peel (*Citrus reticulata* Blanco ‘Cho Khun’) Extract

**DOI:** 10.3390/gels11100808

**Published:** 2025-10-09

**Authors:** Karthikeyan Venkatachalam, Narin Charoenphun, Chawakwan Nitikornwarakul, Somwang Lekjing

**Affiliations:** 1 Faculty of Innovative Agriculture, Fisheries and Food, Prince of Songkla University, Surat Thani Campus, Makham Tia, Mueang, Surat Thani 84000, Thailand; karthikeyan.v@psu.ac.th; 2 Faculty of Science and Arts, Burapha University, Chanthaburi Campus, Chanthaburi 22170, Thailand; narinch@buu.ac.th; 3 Department of Food Science and Technology, Faculty of Agro-Industry, Kasetsart University, Bangkok 10900, Thailand; chawakwan.n@ku.th

**Keywords:** tangerine peel extract, sodium alginate, gel, beads, physicochemical and functional qualities

## Abstract

The effect of varying sodium alginate (SA) concentrations (1%, 2%, and 3%; SA1–SA3) on the encapsulation of tangerine (*Citrus reticulata* Blanco ‘Cho Khun’) peel extract (TPE, 0.5% *w*/*v*) into hydrogel beads was evaluated. Overall, the results showed that increasing SA concentration significantly altered bead characteristics: lightness (L*) decreased from 56.35 to 45.57, red-green axis (a*) shifted negatively from −1.32 to −6.87, and yellow-blue axis (b*) increased from −17.81 to 6.41. Moisture content (97.85% to 93.16%) and water activity (0.96 to 0.93) declined with higher SA, while hardness increased (4.12 to 5.17 g). ζ-potential values shifted from −29.10 mV (SA1) to −39.10 mV (SA3), confirming enhanced electrostatic stabilization. FTIR spectra revealed characteristic alginate functional groups, and morphological analysis showed smoother, denser beads at higher SA concentrations. Phenolic (47.86–48.51 mg GAE g^−1^ DW) and flavonoid (34.02–36.68 mg QE g^−1^ DW) contents were well-retained, supporting antioxidant activities (DPPH 70.34–72.54%; ABTS 65.66–66.91%). Antimicrobial tests demonstrated > 4-log reductions against *E. coli* and *P. aeruginosa*. Sensory evaluation revealed that higher SA concentrations improved texture and taste. Overall, SA encapsulation, particularly at 3%, effectively stabilized TPE, preserving its functional properties for potential food and nutraceutical applications.

## 1. Introduction

Agro-industrial byproducts play a vital role in sustainable food systems and controlling the environmental burdens. Tangerine fruit (*Citrus reticulata* Blanco ‘Cho Khun’) peel constitutes approximately 35–40% of its fruit’s mass [1,2], and predominantly it is considered as waste and discarded during the juice processing, and this adversely generates high biochemical oxygen demand (BOD) in wastewater and contributes to environmental pollution [3]. On the other hand, tangerine peel contains a rich source of bioactive secondary metabolites, particularly hesperidin (1.5–3.2%, dry weight), nobiletin (0.05–0.17%, dry weight), and tangeretin (0.03–0.15%, dry weight), that exhibit various functional properties, including antioxidant, anti-inflammatory, and antimicrobial properties [4,5,6]. In addition to its functional and biochemical properties, the incorporation of tangerine peel into functional foods could control the inherent instability of polyphenols under processing and storage conditions. Generally, thermal treatments, pH fluctuations, and exposure to light and oxygen could adversely induce the degradation pathways and diminish the bioactivity and bioavailability of tangerine peel, and consequently, its therapeutic potential could be adversely compromised [7,8,9]. Specifically, the hesperidin undergoes hydrolytic cleavage under low pH conditions, nobiletin and tangeretin are susceptible to oxidative cleavage when exposed to light and oxygen, and all three are highly sensitive to high-temperature processing, leading to significant loss of bioactivity [10,11]. As a result, valorization of tangerine peel into commercially viable functional ingredients could preserve and control the labile bioactive compounds. Among the valorization processes, tangerine peel extract (TPE) produced via ethanol–water extraction (typical yield of around 10–13% dry basis) is a cost-effective method that utilizes minimal processing and is suitable for a wide variety of incorporations into functional foods [12,13]. The encapsulation technique is a widely adopted strategy in the food industry that entraps the labile bioactive compounds of natural extracts within the protective matrix, thereby reducing degradation, enabling controlled release, and improving dispersion in aqueous systems. In addition, encapsulation can also be able to mask undesirable tastes or colors, enhancing consumer acceptability. For polyphenol-rich plant extracts, encapsulation stabilizes active compounds against oxidation, hydrolysis, and photodegradation, thus extending shelf life and broadening their application in food, nutraceutical, and pharmaceutical industries [14,15].

Several gelators, particularly proteins, carbohydrates, and synthetic polymers, have been widely employed to produce encapsulation; however, among these, the polysaccharide-based matrices are often preferred, especially for food applications, due to their vast biocompatibility and regulatory acceptance. Among the polysaccharides, sodium alginate (SA) is a GRAS-approved sodium salt of alginic acid, which is a naturally occurring anionic polysaccharide extracted primarily from the cell wall of brown seaweeds. SA consists of linear chains of β-d-mannuronic acid and α-l-guluronic acid residues arranged in varying block patterns that determine its physicochemical behavior, particularly its strength, porosity, and release kinetics [16]. SA effectively encapsulates natural bioactive compounds through aqueous methods by forming Ca-induced gels at ambient conditions. SA is widely preferred due to its biocompatibility, biodegradability, safety, inexpensiveness, and effectiveness at protecting and controlling the release of a wide range of bioactive substances [17,18]. SA performance as an encapsulator is highly concentration-dependent, as concentration adjustments alter the hydrogel network density and diffusion rates. These changes directly affect encapsulation efficiency, gel stability, and the release profiles of the encapsulated substances [19,20,21,22]. There are several studies that have individually investigated the functional and nutraceutical application of TPE as well as the encapsulation efficiency and efficacy of SA.

However, there is no study or limited literature covering the full array of physicochemical, structural, functional, and consumer acceptability characteristics specifically for the Cho Khun cultivar that has not been identified. Alginate-based encapsulation is widely used for labile phytochemicals because ionotropic gelation with Ca^2+^ forms hydrated networks that entrap solutes while modulating diffusion [23]. Prior studies show that process route (external ionic extrusion vs. internal gelation), polymer attributes (M/G ratio, concentration), and crosslink density govern bead opacity/turbidity, moisture/aw, mechanical integrity, ζ-potential/colloidal stability, and release kinetics of phenolics/flavonoids [24]. Studies on citrus-derived polyphenols and essential-oil actives report that denser alginate matrices (higher SA or Ca^2+^) typically enhance structural stability and reduce water uptake while slowing active release; however, overly rigid networks can impair sensory perception or functional availability [25]. These trade-offs motivate a matrix-focused evaluation under constant active loading.

To decouple matrix effects from active-dose effects, we kept TPE constant (0.5% *w*/*v*) and varied SA (1–3% *w*/*v*), allowing a clean readout of how alginate concentration alone drives physicochemical (color, moisture, aw), structural (morphology), interfacial (ζ-potential), functional (TPC/TFC, DPPH/ABTS; antimicrobial), and consumer outcomes. This comprehensive, application-oriented profiling supports a practical recommendation on SA level (favoring higher SA for integrity and stability) based on quantitative results reported in the manuscript (e.g., hardness increase and more negative ζ-potential at higher SA). The objective of this study was to evaluate how varying SA concentrations (1–3% *w*/*v*), at a fixed TPE loading (0.5% *w*/*v*), influence the physicochemical, structural, functional, and sensory properties of TPE-loaded gel beads and to identify a formulation suitable for food and nutraceutical applications.

## 2. Results and Discussion

### 2.1. Physicochemical Properties

#### 2.1.1. Color Characteristics and Visual Appearance

**Figure 1:**
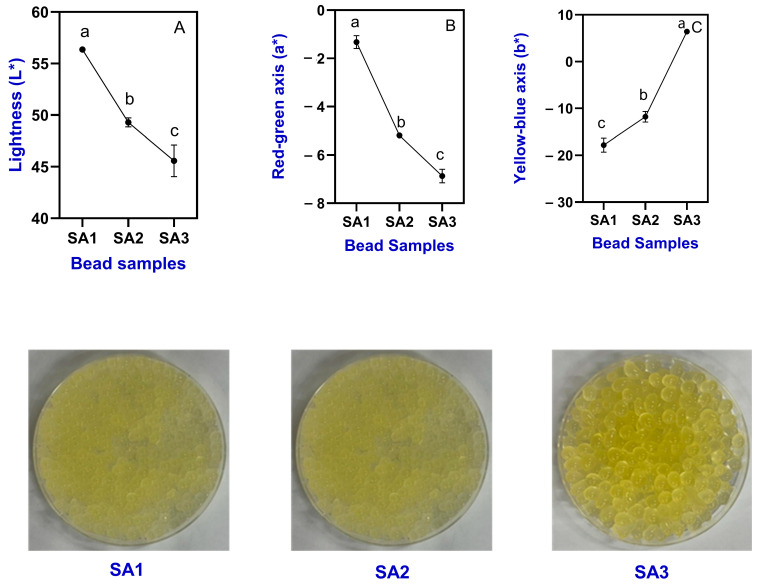
Color characteristics such as lightness (**A**), red-green axis (**B**) and yellow-blue axis (**C**) and visual appearance of TPEen-SA-based gel beads. Different letters in the figure indicate significant differences (*p* < 0.05).

Color is an important sensory attribute as well as an important physical property of food. The present study exhibited linear trends in gel bead color characteristics. An increased concentration of SA had significantly altered the color characteristics (L*, a*, and b*) (Figure 1A–C). The linearity of these changes indicates that the concentration-dependent modification of the gel matrix systematically influenced light scattering and absorption. This predictable trend is important because it demonstrates reproducibility and provides a basis for process control and product quality optimization in encapsulation systems. Among them, the L* value in SA1 samples was 56.35, which gradually decreased to 45.57 as SA concentration increased. This could be due to the increased SA concentration, which resulted in denser alginate matrices that enhanced turbidity and opacity by intensifying light scattering and reducing transparency. Furthermore, higher SA levels may increase water retention by reduced the porosity of the gel and form a thicker gel matrix, which further reduces transparency and lightness (L*). The incorporation of TPE also contributed to lower L* values due to pigments, oils, and solid particles, which increased turbidity and reduced transparency. Dhasmana et al. [26] found that alginate and orange peel extract-based hydrogel beads can alter the microstructure and thus affect the light scattering properties and reduce lightness. Julaeha et al. [27] reported the encapsulation of *Citrus aurantifolia* peel extract into the alginate gelatin microbeads, and thus adversely affected the particle size and surface roughness and contributed to color loss.

On the other hand, red-green axis (a*) values in the TPEen-SA-based gel beads were negatively increased upon increased SA concentration of gels. SA1 samples had a value of −1.32, and it became more negative and exhibited −6.87 in SA3 samples. These results indicate a gradual shift towards green hues. This shift can be explained by the denser alginate network at higher SA concentrations, which disperses pigments more uniformly and increases light scattering, thereby masking red hues and accentuating greenish tones. Generally, SA-based gels tend to have neutral to slightly greenish values, controlled by gel concentration and conditions. This is in accordance with the study of Mostaghimi et al. [28] which observed similar green hues on the alginate-based gel beads. On the other hand, the b* values increased from a strongly negative value in SA1 (−17.81) samples to a moderately positive range (6.41) in SA3 samples. Though the TPE concentration is fixed, the increased SA concentration in gel beads plays a significant role. A sudden color shift was noticed in samples, from a strong blue or yellow-greenish tilt to a more yellow appearance upon increased concentration. It could be due to SA1 samples having less polymer concentration, thus allowing more blue light-based transmission, while SA3 samples cause more scattering and also absorption of shorter wavelengths, leading towards yellow hues [29]. Colin et al. [23] reported that an increase in polymer concentration in the gel beads could affect matrix density and bead microstructure, which alters the pigment distribution and color attributes, particularly b* values, by impacting their light scattering and absorption. Visual observation showed that TPEen-SA-based gel beads at lower concentration (SA1) had exhibited less compact packing with more interstitial space, which indicates low structural rigidity and mechanical stability (Figure 1). Furthermore, the appearance of the gel indicates that it is slightly flattened and irregular in shape. A clear correlation was observed between SA concentration and bead morphology: at lower SA (SA1), weaker gelation and lower rigidity led to flat and irregular shapes, while at higher SA (SA2–SA3), stronger crosslinking and denser gel networks produced smoother, more spherical beads. This correlation is also visible in the stereomicroscopy images of this study. On the other hand, the increased concentrations, such as the SA2 and SA3 samples, were more of a uniformity and spherical geometry. It indicates that increased SA concentration could improve spatial organization and reduce deformation. Furthermore, SA2 samples exhibited more tightly packed gel beads than the other samples, and it is suggested that enhanced mechanical resistance and internal cohesion could be due to the strong cross-linking effect by the alginate network. This is in accordance with the study of Xiao et al. [30], who suggested that SA-based gel beads, especially at a higher concentration, can be able to produce more uniform and tightly packed beads. Kowalski et al. [31] reported that increased SA concentration induces cross-linking followed by increased water uptake, structural integrity, and internal compactness of the hydrogel and thus leads to increased spatial arrangement and mechanical stability.

#### 2.1.2. pH and TSS

**Figure 2:**
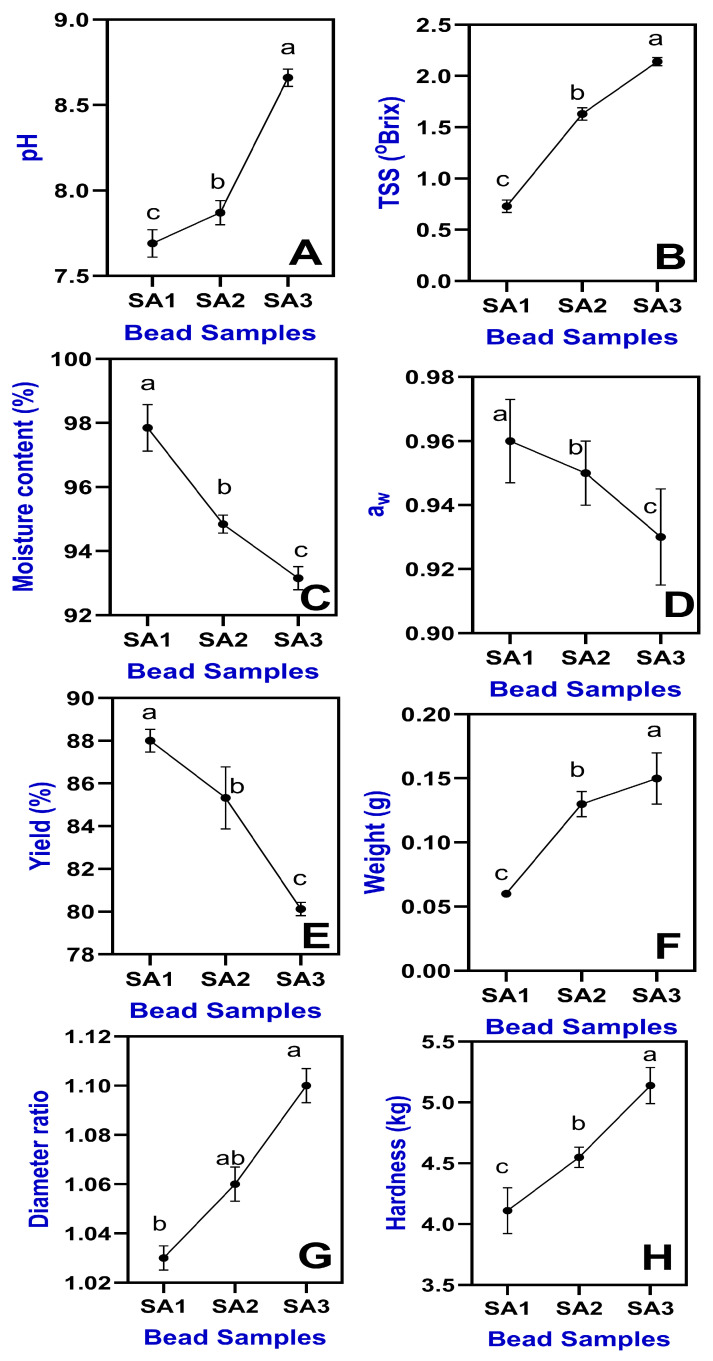
Changes in pH (**A**), TSS (**B**), moisture content (**C**), a_w_ (**D**), yield (**E**), weight (**F**), diameter ratio (**G**) and hardness (**H**) of TPEen-SA-based gel beads. Different letters in the figure indicate significant differences (*p* < 0.05).

pH modulates the SA-based gel beads’ stability by altering alginate ionization and the integrity of divalent-cation crosslinks. This study found that the pH level of all tested TPEen-SA-based gel beads ranged between 7.69 and 9.66 (Figure 2A,B). pH of the samples was significantly influenced by SA concentration. The higher concentration of SA had increased pH to slightly alkaline in beads. Whereas the lower SA concentration-based samples keep the pH of beads slightly above neutral. This study indicates that TPE might have slightly influenced the pH level in beads. This study indicates that TPE might have slightly influenced the pH level in beads. However, these values are still well below the reported zero-point charge of sodium alginate, which is approximately pH 7, indicating that the carboxyl groups remained deprotonated and negatively charged under the tested conditions, which supports the electrostatic stability of the bead matrix. Studies have reported that the pH of aqueous TPE was around 3–5, and beads significantly higher than this level indicate that SA played a significant role, and also TPE would usually be mildly acidic. Furthermore, commercial SA often contains residual sodium salts, and it could be the reason for the rise in pH in the tested bead samples. Furthermore, increased SA in the beads’ composition might contribute to increased sodium salts and thus elevate the beads’ pH [32]. However, studies suggest that the alkaline pH of beads could increase swelling, soften, and degrade the ionically crosslinked SA-based gel beads as well as reduce the mechanical integrity [33]. This behavior can be explained by the greater ionization of alginate carboxyl groups under alkaline conditions, which enhances electrostatic repulsion between polymer chains. As the SA gel network expands, water uptake and swelling increase, but the ionic crosslinks weaken, thereby reducing mechanical integrity. Similar mechanisms have been reported in the study of Malektaj et al. [34] on SA-based hydrogel. TSS content in TPEen-SA-based gel beads that encapsulated TPE and were made with different SA concentrations is shown in Figure 2. Overall, the TSS levels in the tested samples steadily increased with increased SA. Although the samples exhibited an increasing trend, the TSS levels did not exceed more than 2. A linear increase in TSS level was found among the samples. Makarova et al. [35] reported that increased gelator concentration in the composition could induce the increase in network density, which effectively immobilizes the water within junction zones and interstitial spaces, and thus ultimately reduces the free water and concentrates the dissolved species and consequently elevates the observed TSS level. Łętocha et al. [36] found that increased alginate enhances extensive cross-linking and denser alginate matrices and reduces polymer leaching, retaining more alginate within the gel and thereby increasing levels in the gelled products.

#### 2.1.3. Moisture Content and Water Activity

Moisture content and water activity of the gel bead samples decreased continuously with increasing alginate concentration (Figure 2C,D). Moisture results showed that SA1 samples retained a higher level of total moisture (97.85%), and it gradually decreased to 93.16% in the SA3 samples. Similarly, the water activity levels in the tested gel bead samples decreased with increased SA concentration; the aw ranged between 0.96 and 0.93. Increased SA concentration could densify the hydrogel network and also increase the water binding capacity, thus reducing the proportion of free and mobile water [37,38]. Savic et al. [39] reported that alginate and CaCl_2_ help water retention and swelling capacity in the beads and consequently lower free water. This decrease can also be explained in terms of alginate’s hydrophilic functional groups (–OH and –COOH), which form hydrogen bonds with water molecules. At higher SA concentrations, more binding sites are available, leading to stronger retention of bound water and a lower proportion of free water, thereby reducing both moisture content and a_w_. This discreetly improves the shelf life of beads by enhancing physical stability, by limiting syneresis and diffusion of oxygen, and consequently produces firmer and less sticky beads [40].

#### 2.1.4. Weight and Yield

Different concentrations of SA significantly increased the weight of TPE-encapsulated beads (Figure 2E,F). Bead weight ranged from 0.06 to 0.15 g. The difference between SA1 and SA3 was significant; however, SA2 did not differ much from SA3. This indicates that the amount of polymeric material in the gel matrix increased with SA concentration. In general, increasing SA concentration increases the wet weight of ionotropically gelled beads, in accordance with Bennacef et al. [41]. Higher SA concentrations can increase the viscosity of the bead-forming solution, promoting the formation of larger droplets during dripping or extrusion and thus larger beads with greater mass [42]. Aldawsari et al. [43] reported that higher alginate concentrations allow more polymer chains to crosslink with Ca^2+^, creating a denser network with greater polymer mass, higher entrapment efficiency, and reduced leaching of encapsulated actives. On the other hand, the production yield of TPEen-SA-based gel beads declined progressively from 88% to 80% as the SA concentration increased. The increase in SA concentration promoted superior physical integrity, particularly in the SA3 samples, which led to higher hardness and lower water availability and thereby adversely affected yield recovery [36,42,44]. In general, higher alginate concentrations favor more viscous and faster interfacial gelation, which reduces droplet detachment efficiency, increases tailing at the gelling surfaces, and elevates losses during sieving and drying. All of these factors contribute to a lower net yield [23,36].

#### 2.1.5. Diameter Ratio and Hardness

The diameter ratio of TPEen-SA-based gel beads that encapsulate TPE and are made with different SA concentrations is shown in Figure 2G. The results exhibited that increasing SA concentration non-significantly increased the diameter ratio, which ranged between 1.03 and 1.10. Higher SA concentration might increase feed viscosity and slow ion exchange during gelation, causing greater osmotic uptake before network lock-in, which reduces gel syneresis and consequently increases the diameter ratio [45]. Although the change in diameter ratio was not statistically significant, Colin et al. [23] reported that a larger diameter ratio generally enhances mechanical stability and encapsulation efficiency, leading to slower and more sustained release of the encapsulated material. Similarly, the hardness values in the tested TPEen-SA-based gel beads were stronger with increased SA concentration (Figure 2H). Among the samples, SA3 showed significantly higher hardness compared to the lower concentrations (SA1 and SA2). This could be due to the formation of a denser polymer network formed at higher SA concentration, which strengthens cross-linking with Ca^2+^ ions [46]. Studies have reported that increased hardness improves mechanical resistance as well as reduces the deformation under stress, which enhances the handling stability during storage and application [47]. In addition to the role of SA concentration, the incorporation of TPE also contributed to the mechanical properties of the beads. The polyphenolic compounds in TPE are likely to interact with alginate chains via hydrogen bonding and secondary interactions, which reinforce the gel network. This interaction effect, together with the denser polymer structure at higher SA levels, further enhanced bead hardness and reduced deformability. Similar trends have been reported in previous studies, where higher SA concentrations yielded firmer, more rigid beads due to increased junction zone formation and tighter polymer packing [41].

### 2.2. Structural and Molecular Characteristics

#### 2.2.1. FTIR

**Figure 3:**
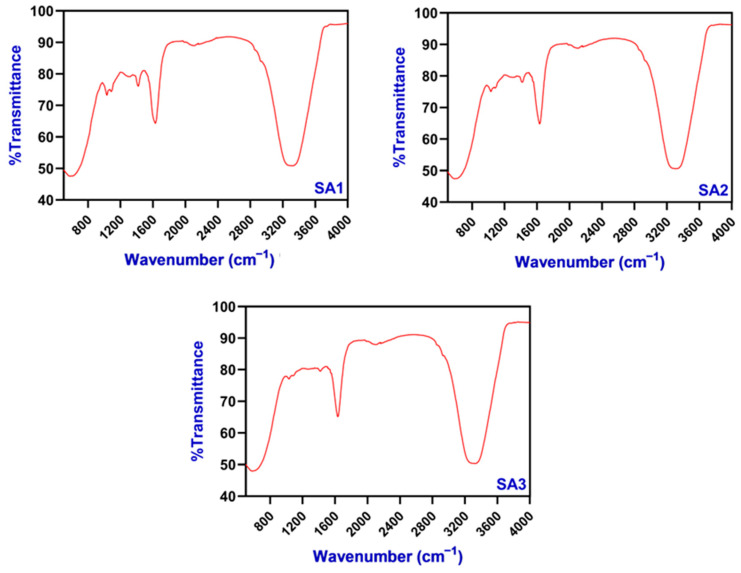
Differences in FTIR spectra of TPEen-SA-based gel beads.

FTIR spectra of edible TPEen-SA-based gel beads that encapsulate TPE and are made with different concentrations of SA are shown in Figure 3. The results showed the spectra in all the treatments at different wavenumbers. A spectral peak at ~3200–3600 cm^−1^ was found in all treatments, indicating broad O-H stretching bands, representing water and polysaccharide hydroxyl groups. Belattmania et al. [48] characterized the SA from brown seaweeds and identified similar spectral bands at 3200–3400 cm^−1^ corresponding to O-H stretching. A slight peak was noticed at the wavenumber of 2900 cm^−1^, especially at higher concentrations of SA, indicating a C-H stretching vibration, which also acts as a backbone for the alginate sugar ring [49]. A prominent steep peak was found at 1600 cm^−1^ and 1400 cm^−1^ in all the samples, but the intensity of the peak was high in SA2 and SA3 samples, representing asymmetric and symmetric carboxylate (COO^−^), indicating a stretching of alginate. Furthermore, these functional groups are critical for ionic cross-linking and gel network formation, and their intensities also correlate with gel stretching [50]. The C-O stretching was also prominent at near 1040 cm^−1^, indicating the presence of polysaccharides [48,50]. Overall, the multipoint spectral observation represents the effective formation of SA gel beads and the presence of bioactive compounds from the added natural extracts. The variation in peak spectra among samples, particularly in the O-H region, represents the water-holding capacity of treatments. However, there are no specific FTIR spectra found for TPE, indicating that the added extract at fixed concentrations does not contribute to functional groups, or their signal is likely to be masked or overlapped by the alginate matrix. Khorshidian et al. [51] reported that herbal extract loaded with alginate-based microcapsules exhibited FTIR peaks within the alginate region, and it was mostly detected indirectly via changes in alginate peak shape or intensity variation, and their study concluded that due to the low concentration of the herbal extract, it was unable to develop a new peak or band on the FTIR spectra. This is in accordance with the study of Savic et al. [39] and Stanislawska et al. [52].

#### 2.2.2. Morphological Observations

**Figure 4:**
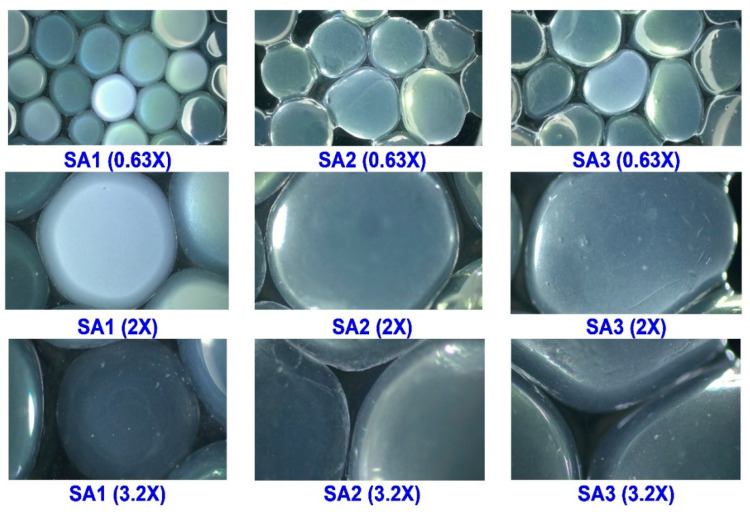
Morphological observation of TPEen-SA-based gel bead samples.

**Figure 5:**
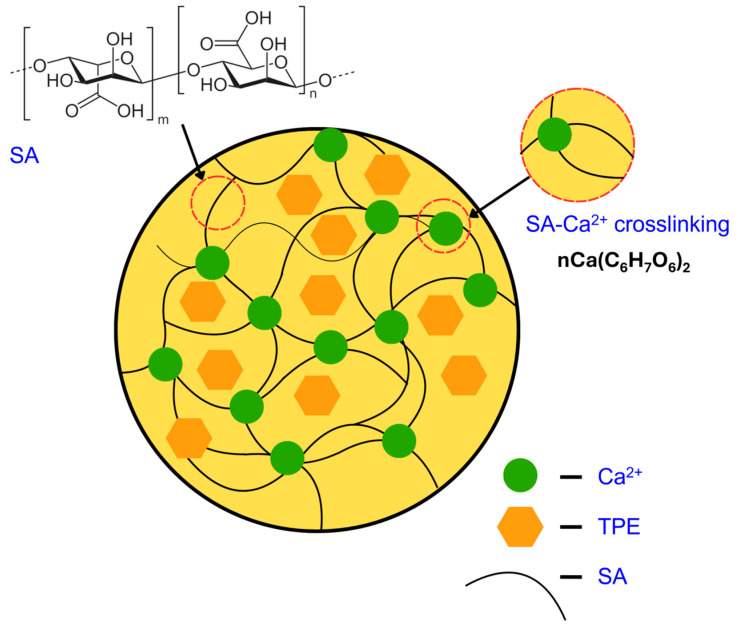
Schematic representation of SA–Ca^2+^ crosslinking (egg-box model) and encapsulation of tangerine peel extract (TPE). Alginate chains provide carboxylate groups (–COO^−^) that coordinate with Ca^2+^ ions, forming junction zones that strengthen the hydrogel network. TPE molecules are entrapped and stabilized by the dense matrix through hydrogen bonding and secondary interactions.

Morphological observations of TPEen-SA-based gel beads that encapsulate TPE and were made with different SA concentrations are shown in Figure 4. In general, the morphological observations of TPEen-SA-based gel beads indicate integrity, uniformity, compactness, and smoothness. Among the tested samples, SA1 beads showed less defined boundaries and moderate translucency. On the other hand, the SA2 and SA3 samples exhibited smoother surfaces, better-defined boundaries, and consistent translucency. Encapsulation of TPE also contributed to these morphological outcomes, as interactions between bioactive compounds and alginate chains enhanced compactness and reduced porosity. These effects were more pronounced at higher SA concentrations, resulting in smoother and more spherical beads. Galogahi et al. [53] found that increased alginate concentration could increase the viscosity of the solution and support more uniform and spherical droplets. Increased SA concentration tightens the network of cross-linking and thus expels the water molecules, resulting in compact beads with smooth external surfaces [54,55]. Furthermore, SA3 samples also demonstrated minimal interstitial voids and well-rounded contours compared with the other samples, indicating an optimal level of gelation and a denser matrix. Studies have reported that higher SA concentration induces a higher degree of polymer interactions and cross-linking, which occupies the interstitial space of beads and produces dense and well-rounded contours [41,56]. The observed increase in compactness and smoother surfaces at higher SA concentrations can be explained by the classical egg-box model of alginate crosslinking. As illustrated in Figure 5, Ca^2+^ ions coordinate with –COO^−^ groups of alginate chains to form ionic junctions, tightening the polymer network and reducing void spaces. TPE molecules are entrapped within this matrix and stabilized through hydrogen bonding and secondary interactions, consistent with the denser, more spherical beads observed microscopically.

#### 2.2.3. Swelling Capacity and Encapsulation Stability

**Figure 6:**
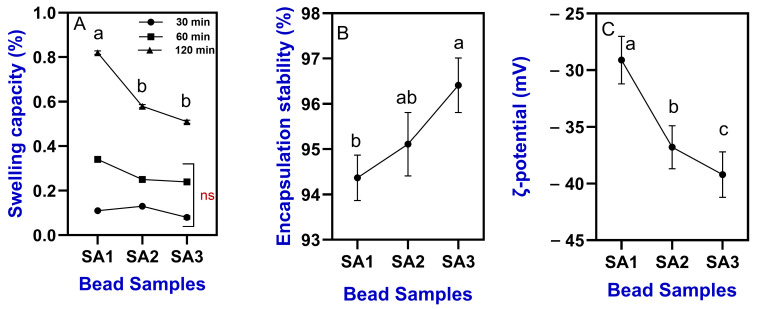
Changes in swelling capacity (**A**), encapsulation stability (**B**), and ζ-potential (**C**) of TPEen-SA-based gel beads. Different letters in the figure indicate significant differences (*p* < 0.05).

The swelling capacity of TPEen-SA-based gel beads is shown in Figure 6A. The swelling capacity was tested at different durations on gel beads to explore the dynamic water uptake behavior of SA concentration. Among the samples, SA3 exhibited the lowest swelling capacity, despite the increased SA concentration. Overall, the increased duration significantly increased the swelling capacity of the tested beads. At 30 min, the swelling capacity was 0.11%, 0.13%, and 0.08% for SA1, SA2, and SA3, respectively, and when the duration was increased to 120 min, the results were 0.82%, 0.58%, and 0.51%, respectively. Among the samples, SA1 showed more immediate swelling at a short duration (30 min); however, it plateaued at lower levels. The results indicate that increased concentrations of SA showed high initial resistance to water penetration, which could be due to tighter cross-linking and is very favorable for controlled-release applications. This apparent contradiction can be explained by the denser gel network formed at higher SA concentrations, which reduces pore size and restricts rapid diffusion of water molecules during the initial stages of swelling. Although alginate contains abundant hydrophilic groups, many of these sites become engaged in Ca^2+^ crosslinking, leaving fewer available for immediate water binding. As a result, initial penetration is slower, even though bound water uptake continues over longer times. Studies have reported that a decrease in swelling properties with increased alginate concentration could be due to the alginate filling of the void spaces in the gel bead network, which would otherwise be available to water molecules, and this could adversely limit the penetration of water molecules into the gel network [31,33]. Agles and Bourg [37] and Savić Gajić et al. [57] reported that increased alginate concentration induces calcium ion–induced cross-linking that imparts resistance to water penetration in the gel beads. The encapsulation stability of TPEen-SA-based gel beads is shown in Figure 6B. Overall, the trend showed that increased SA concentrations significantly affected the encapsulation stability. Among the samples, higher SA concentrations effectively stabilized the encapsulation compared with lower concentrations. This indicates that higher SA concentration can enhance long-term stability through reduced synergies by minimizing water expulsion over time [58] and resistance to degradation by increased cross-linking density, which reduces the enzymatic and chemical degradation of encapsulated products [42]. Sankalia et al. [59] demonstrated that increased SA-based polymer concentration could result in a great increase in the particle strength of gel beads as well as enhanced entrapment efficiency. Several studies have reported that increased SA concentration constrained the enlargement of pore size within the gel network and thus improved the retention of encapsulated materials and reduced the diffusion rate of degradation agents [33,60].

#### 2.2.4. Zeta (ζ) Potential

ζ-potential values of TPEen-SA-based gel beads are shown in Figure 6C. Overall, the trend exhibited that increasing the SA concentration in the bead formulation had led to progressively retaining more negative ζ-potential values in the samples. The values ranged between −29.10 and −45.20 mV. SA3 had the most ζ-potential values. The results showed a monotonic decrease in the bead samples with increased SA concentrations, indicating the higher surface coverage by the deprotonated alginate carboxylates, which might increase the diffuse layer and electrostatic repulsion potential. Consequently, the bead’s colloidal electrostatic stabilization increases with SA content, transitioning from a moderate level of ζ-potential values (−30 mV, SA1) to a strong one (−45 mV, SV3). Generally, ζ-potential is very sensitive to pH, ionic strength, and counterion valency [61]. Studies have reported that an increase in SA in the bead composition could raise the number of -COO- groups at the interface and above the alginate’s pKa, which are negatively charged and thus increase the charge density and exhibit an increased negative value [62,63]. Amin and Boateng [64] reported that higher SA levels form a denser and/or more continuous alginate layer around the particles, exposing more anionic sites to the liquid and slowly shifting the diffuse-layer potential towards negative values. Studies reported that more negative ζ-potential values could induce repulsive force between the beads and lower the collision-based aggregation and help beads remain discrete during processing and storage. Furthermore, the negative surface of beads provides a better barrier against handling [65,66]. The richness of deprotonated alginate carboxylates helps tighten the polyelectrolyte network at the bead surface, reducing pore size and diffusivity. Consequently, it supports the higher encapsulation efficiencies observed as SA increases and can slow the release of entrapped actives [23,60]. Furthermore, a more negative bead surface can repel weakly cationic contaminants and some proteins, helping maintain bead surface cleanliness and performance consistency [67].

### 2.3. Phytochemicals and Antioxidant Properties

**Figure 7:**
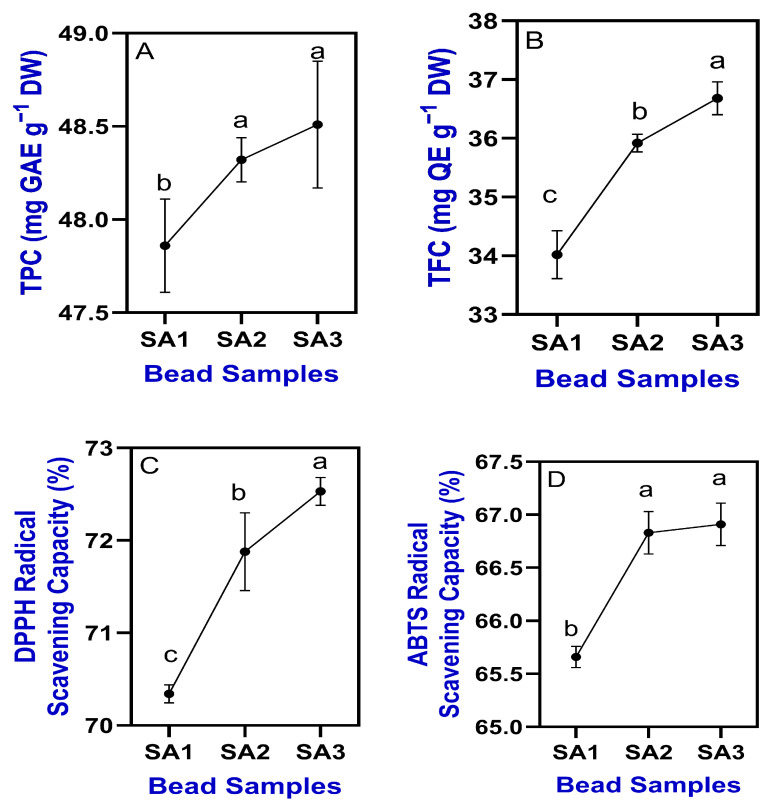
Changes in TPC (**A**), TFC (**B**), DPPH radical scavenging activity (**C**) and ABTS radical scavenging capacity (**D**) of TPEen-SA-based gel beads. Different letters in the figure indicate significant differences (*p* < 0.05).

Phytochemicals such as TPC and TFC in the tested TPEen-SA-based gel beads are shown in Figure 7A,B. Overall, the results indicate that the contribution of phytochemical and antioxidant properties is mainly attributed to TPE [68], while the SA-based gel beads played a significant role in their retention. The TPC in the TPEen-SA-based gel beads ranged between 47.86 and 48.51 mg GAE g^−1^ DW. Similarly, the TFC ranged between 34.02 and 36.68 mg QE g^−1^ DW. TPC and TFC results showed that increasing SA concentration in the gel bead formulation led to higher retention levels. However, the differences between SA2 and SA3 in terms of phytochemical levels were not statistically significant (*p* ≥ 0.05), whereas both differed significantly from SA1. Increasing SA concentration may form a stable Ca–SA gel network through strong ionic cross-linking, which creates a protective barrier and consequently minimizes oxidative and structural degradation of the bioactive compounds. Previous studies have also shown that SA provides a strong protective matrix for phenolic compounds [69]. The antioxidant capacity, assessed by DPPH and ABTS radical scavenging activity of TPEen-SA-based gel beads, is shown in Figure 7C,D. Overall, the antioxidant capacity of the tested samples was consistent with their phytochemical content. As SA alone lacks intrinsic antioxidant activity, the functional effects observed here can be attributed to the incorporated TPE. Increasing SA concentration primarily modified the gel matrix, thereby influencing encapsulation efficiency, stability, and the release behavior of TPE. The DPPH radical scavenging activity of the bead samples showed a slight increasing trend, ranging from 70.34% to 72.53% with increasing SA concentration.

Similarly, the ABTS radical scavenging activity also exhibited an increasing trend; however, the differences between SA2 and SA3 were minimal. The ABTS activity of the samples ranged between 65.66% and 66.9%, demonstrating a positive correlation with SA concentration. Overall, the results demonstrate that the antioxidant capacity of the SA-based gel beads samples containing encapsulated TPE was successfully maintained. This finding is particularly significant because, compared with the free form of antioxidant compounds, encapsulation preserved a greater proportion of antioxidants, which is in accordance with the findings of Jing et al. [70]. Antioxidant compounds in plant extracts are generally very sensitive to environmental factors such as light, oxygen, and pH changes; however, the SA matrix provides protection when encapsulated [20,44,71], which is crucial for maintaining the biological efficacy of the extracts during storage and application. Among the tested SA formulations, the SA3 samples exhibited consistent performance, optimal structural integrity of the beads, and enhanced protection of the encapsulated bioactive compounds. Several studies have shown that SA concentration and crosslinking play a crucial role in the encapsulation efficiency and stability of bioactive compounds [20,72]. This is particularly critical for pharmaceutical and nutraceutical applications, where maintaining bioactivity during processing, storage, and delivery is essential. Although SA3 performed better than the other formulations in retaining both phenolic content and antioxidant activity, all three formulations confirm the suitability of SA as an encapsulation matrix for preserving bioactive compounds from plant extracts.

### 2.4. Antimicrobial Activities

**Figure 8:**
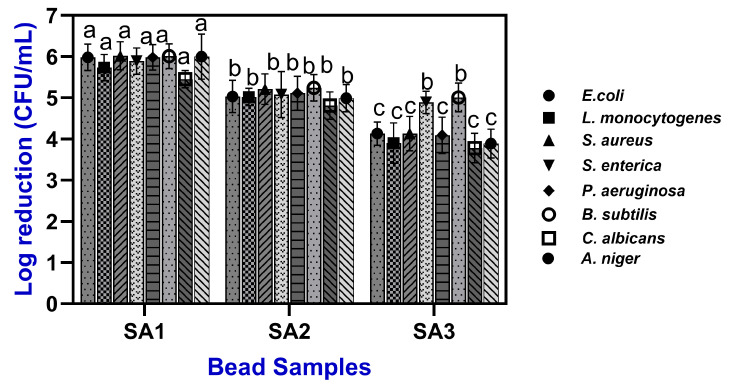
Antimicrobial activity of TPEen-SA based gel beads. Different letters in the figure indicate significant differences (*p* < 0.05).

The antimicrobial activities of TPEen-SA-based gel beads are shown in Figure 8. The test results exhibited that TPE in the gel beads was effective in controlling both pathogens and spoilage microorganisms. Several studies have proven that citrus-based fruits, particularly tangerine peel, are highly effective for microbial attenuation [73,74,75]. The tested microorganisms included *E. coli* (7.2 ± 0.35 log CFU/mL), *S. aureus* (7.0 ± 0.28), *L. monocytogenes* (6.5 ± 0.53), *S. enterica* (6.8 ± 0.93), *P. aeruginosa* (7.1 ± 0.38), *B. subtilis* (6.9 ± 0.23), *C. albicans* (6.3 ± 0.43), and *A. niger* (6.0 ± 0.65) as initial microbial loads in the untreated controls. These baseline levels provide the reference for evaluating the inhibitory capacity of the TPEen-SA-based gel beads in terms of log reductions. Although the TPE concentration in the beads was fixed (0.5%), the antimicrobial activity of the beads demonstrated a dose-dependent inhibition, and it was mainly related to the SA concentrations. In comparison with SA2 and SA3 samples, the SA1 samples exhibited the lowest reductions, ranging from 5.28 ± 0.31 to 5.98 ± 0.32 logs across the tested strains. This effect was enhanced in the SA2 samples, which showed reductions of 4.51 ± 0.34 to 5.22 ± 0.37 logs, while the SA3 samples consistently exhibited the strongest activity, with reductions exceeding 4 logs, particularly against *E. coli* (4.13 ± 0.39 logs) and *P. aeruginosa* (4.09 ± 0.44 logs). Reductions were also high for *S. aureus* (4.13 ± 0.42 logs) and *S. enterica* (4.00 ± 0.37 logs). Rijo et al. [76] tested the encapsulation of plant extract into SA beads and found high loading with effective antimicrobial activity; because alginate concentration modulates bead structure and release, this framework is consistent with stronger inhibition at higher alginate concentrations even when extract loading is constant. Sabry et al. [73] reported that orange and tangerine peel extracts showed greater antimicrobial reductions as SA concentration increased, despite a fixed extract loading in the beads. Among the tested strains, *C. albicans* (3.78 ± 0.36 logs) and *A. niger* (3.89 ± 0.35 logs) were relatively more resistant. This is in accordance with the study of Tøndervik et al. [77], who reported that alginate-based materials and alginate oligosaccharides inhibit fungi but yield smaller effects than bacteria. Overall, these findings suggest that TPE encapsulated with SA gel beads not only exerts inhibitory effects on foodborne pathogens but also suppresses typical bacterial, yeast, and mold contaminants, which ensures food safety and shelf-life endurance.

### 2.5. Sensory Characterization

**Figure 9:**
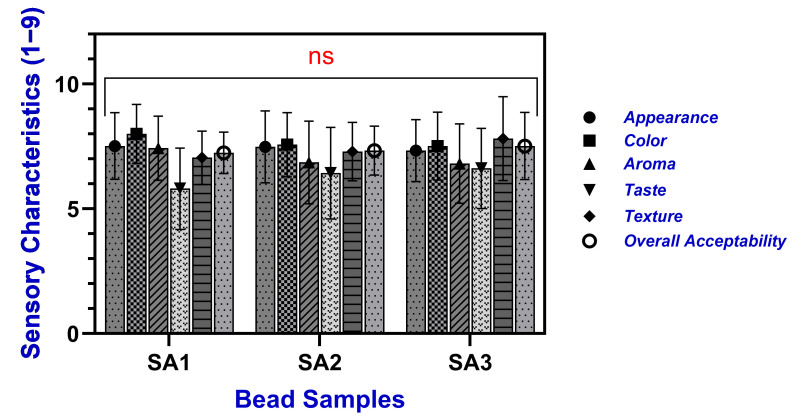
Sensory characteristics of TPEen-SA *based gel beads*. Different columns represent sensory attributes; ns denotes no significant difference (*p* ≥ 0.05).

The sensory qualities data of TPEen-SA-based gel bead samples reveals the complex relationship between SA concentration and consumer acceptability. Overall, the findings from the sensory characteristics demonstrate that higher SA concentration had significantly affected the TPE-encapsulated beads in terms of visual appeal and odor (Figure 9). Higher SA concentration had lowered the appearance score of gel beads from 7.52 to 7.33, despite the small differences between the SA samples. Also, the color score declined with increased SA concentration, which is consistent with instrumental findings. This is in accordance with the study of Aguirre-Calvo et al. [78], who tested the alginate bead in different foods. However, the color scores did not vary significantly between the SA2 and SA3 samples. Sobri et al. [79] also found similar results. Overall, the color scores were decreased with the increased SA concentration; however, the increasing SA concentration did not affect much on the sample’s color characteristics. Stribicaia et al. [80] reported that higher SA concentration in the gel beads creates a denser polymer network during crosslinking and thus adversely affects the visual characteristics. Similarly, the trend in odor values was comparable to that of appearance and color scores. Higher SA concentration had an adverse effect on the aroma quality of the beads. Farahani et al. [81] reported that increasing SA concentration could adversely affect the diffusion of aroma compounds to the headspace, thus diminishing the perceived odor intensity. Furthermore, Uzokboev et al. [82] reported that the lower aroma score in the dense alginate beads is due to the strong entrapment of volatile compounds within the gel structure, thus preventing their release during mastication and reducing overall aroma impact. On the other hand, as SA concentrations increased, taste and texture scores also increased. Sunarharum et al. [83] reported a similar finding that when SA concentration increases in the beads, it consistently makes the gel beads firmer and chewier. The taste and texture were significantly changed with higher SA concentrations. The overall acceptability score demonstrates that consumers’ preference for increased SA concentration, which enhanced the beads’ taste and texture, significantly influenced their choices. However, several studies have suggested that taste and overall acceptance of the gel beads are correlated with the optimum SA concentration rather than the monotonic increase in SA in the gel-based food samples [79,83].

## 3. Conclusions

This study demonstrated that SA-based gel beads are an effective system for encapsulating TPE, allowing for the retention of its bioactive properties while enhancing the structural and functional characteristics of the beads. Increasing SA concentrations improved bead integrity, moisture retention, hardness, swelling capacity, and encapsulation efficiency. FTIR analysis confirmed the physical entrapment of TPE without chemical interaction. Antioxidant and antimicrobial activities of the encapsulated extract were maintained, supporting the functional potential of the encapsulation system. Sensory analysis revealed favorable consumer responses in terms of appearance, texture, taste, and overall acceptability. Although a direct comparison with a non-encapsulated extract was beyond the scope of this study, the use of SA, an inert encapsulating material with no inherent antioxidant or antimicrobial activity, supports the interpretation that the observed bioactivities originated from the encapsulated TPE. These findings highlight the potential of SA-based gel beads as carriers for citrus-derived bioactives in functional food applications.

## 4. Materials and Methods

### 4.1. Chemicals and Reagents

All chemicals and reagents used in this study, including Folin–Ciocalteu reagent, sodium carbonate, gallic acid, sodium nitrite, sodium chloride, aluminum chloride, sodium hydroxide, quercetin, DPPH, ABTS, ethanol, and sodium acetate, were of analytical grade (from Sigma-Aldrich, Bangkok, Thailand) unless otherwise stated. Sodium alginate, calcium chloride, and glycerol were of food grade and purchased from Chemrish Limited Partnership (Bangkok, Thailand). Dey/Engley neutralizing broth and culture media (Mueller–Hinton agar, Sabouraud Dextrose Agar, and Potato Dextrose Agar) were obtained from Himedia, Mumbai, India.

### 4.2. Preparation of TPE

**Figure 10:**
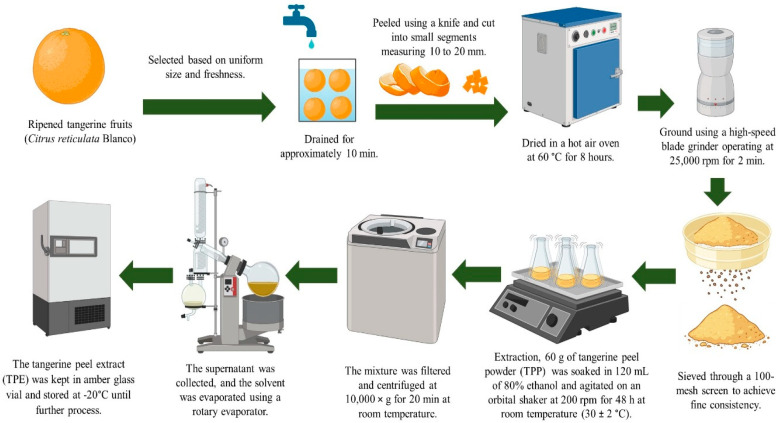
Infographic representation of the preparation of TPE.

Fully ripened tangerines (*Citrus reticulata* Blanco ‘Cho Khun’) with uniform size and optimal freshness were acquired from a local fruit market in Surat Thani Province, Thailand. Then, the fruits were transported to the laboratory and thoroughly examined for any visible damage and were thoroughly washed with distilled water, drained, and dried to remove any residual surface moisture. Following that, the fruits were manually peeled with a knife and cut into small segments (roughly 10–20 mm). Then, the pericarp pieces were dried in a hot air oven (Binder, model FD 115, Tuttlingen, Germany) at 60 °C for 8 h. After that, the samples were cooled to ambient temperature and finely ground into powder using a high-speed blade grinder (Thai grinder, WF-20B, Bangkok, Thailand) operating at 25,000 rpm for 2 min. The resulting tangerine peel powder (TPP) was sieved thoroughly with a 100-mesh screen to achieve fine consistency. Then, TPP was extracted following a modified method from Sharma et al. [84]. In brief, 60 g of TPP was soaked in 120 mL of 80% ethanol. Subsequently, the solution underwent agitation via an orbital shaker (Miulab GS-20, Zhejiang, China) at 200 rpm for 48 h at 30 ± 2 °C. Then, the mixture was filtered and centrifuged at 10,000× *g* for 20 min at ambient conditions. The supernatant as TPE was collected, and the solvent volume was reduced to approximately one-fifth of the initial volume by using a rotary evaporator (Buchi R-300, Flawil, Switzerland), and it was kept in an amber glass vial and stored at −20 °C until further processing. An infographic of the preparation of TPE is given in Figure 10.

### 4.3. Preparation of TPEen-SA-Based Gel Beads

**Figure 11:**
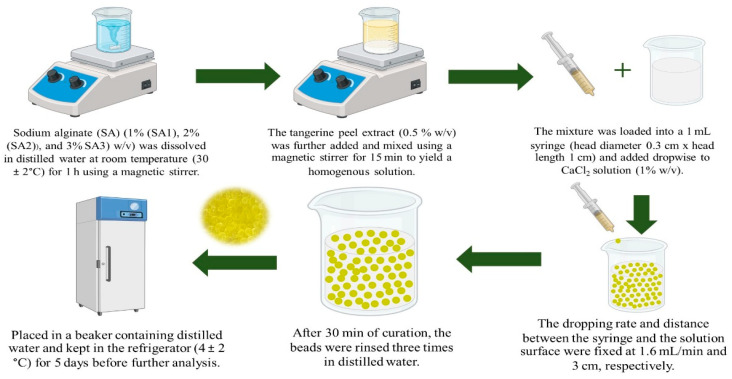
Infographic representation of the preparation of TPEen-SA based gel beads.

The encapsulation procedure was performed according to the method described by Wongverawattanakul et al. [20], with some modifications. SA at concentrations of 1% (SA1), 2% (SA2), and 3% (SA3) (*w*/*v*) was solubilized in distilled water at room temperature (30 ± 2 °C) for 1 h using a magnetic stirrer (C-MAG HS7, IKA, Staufen, Germany). Subsequently, TPE at a fixed concentration (0.5% *w*/*v*) was introduced and combined thoroughly with a magnetic stirrer for 15 min, resulting in a homogeneous solution. For the development of TPE beads, the mixture was transferred into a 1 mL syringe (head diameter 0.3 cm × head length 1 cm) and added dropwise to a 1% *w*/*v* CaCl_2_ solution. The flow rate and the distance from the solution surface were set to 1.6 mL/min and 3 cm, respectively. After that, the beads were kept for 30 min of curation, then the beads were collected and rinsed three times with distilled water, submerged in a beaker containing distilled water, and stored in a refrigerator (4 ± 2 °C) for five days prior to subsequent analysis. An infographic of the preparation of TPEen-SA-based gel beads is given in Figure 11.

### 4.4. Determination of Physicochemical Properties

#### 4.4.1. Color Characteristics and Visual Appearance

The color attributes, including lightness (L*), red-green axis (a*), and yellow-blue axis (b*), were evaluated in the bead samples using a Hunter Lab colorimeter (model AMT501, Jedto, Pathum Thani, Thailand). Prior to analysis, the colorimeter was calibrated against a white reference tile before each session. For each treatment, five beads were measured, with three readings per bead. The visual appearance of the samples was recorded using a handheld digital camera (Coolpix B500, Nikon, Tokyo, Japan).

#### 4.4.2. pH and Total Soluble Solids (TSS)

The pH values of the bead samples were measured with a calibrated tabletop pH meter (Mettler-Toledo GmbH, Giessen, Germany) using pH 4 and pH 7 standards before analysis. For pH, beads were homogenized (1:10 *w*/*v*, bead: DI water) and equilibrated 60 s before reading; the probe was rinsed and blotted between samples. The TSS levels in the bead samples were measured by using a handheld hand refractometer (ATAGO Model PAL-1, Tokyo, Japan, Brix range 0–32%). TSS was measured on the expressed liquid after gentle pressing of beads, with the refractometer prism cleaned and dried between readings. The results are expressed as brix (◦).

#### 4.4.3. Moisture Content and a_w_

The moisture content of the bead samples was determined using an infrared moisture analyzer (Sartorius, MA160, Göttingen, Germany). 2 g of samples were placed on the analyzer, and the operating temperature was set at 105 °C. Moisture was determined in triplicate per treatment using the device’s constant-weight (auto-end) program. The results were reported as percentage (%). The a_w_ of the bead samples was measured at 25 °C using a dew point water activity analyzer (Series 4TEV, Aqua Lab, Pullman, WA, USA). The results were reported as a_w_.

#### 4.4.4. Weight and Yield

The weight of the bead samples was determined by collecting ten beads from each variable group and weighing them using a four-decimal-place analytical balance (Sartorius, Quintix 224-1S, Göttingen, Germany). The mean weight was subsequently calculated and expressed as weight in gram. The yield of the bead samples was calculated based on the weight of the gel beads (in grams) per 100 mL of the SA-based TPE solution and expressed as percentage (%).

#### 4.4.5. Diameter Ratio and Hardness

The diameter of bead samples was determined by following the method of Kamprawet and Vatthanakul [85]. Ten beads of gel bead samples were collected, and the size was measured individually using a Vernier caliper (Mitutoyo, 530-312, Kawasaki, Japan). Bead’s larger diameter, smaller diameter, and the ratio of both diameters were calculated to evaluate the sphericity of the beads, with ratios closer to 1 referring to higher sphericity. The results were expressed as diameter ratio. The hardness of the bead samples was measured using a texture analyzer (CT3, AMETEK Brookfield, MA, USA) according to the method of Phawaphuthanon et al. [86] with modifications. A cylindrical probe (TA10, 25.4 mm diameter) was used at a crosshead speed of 1 mm/s with a maximum load of 10 N. Each bead was compressed at 25 ± 2 °C until fracture, and hardness was defined as the maximum compression force recorded at that point. Twenty individual beads per treatment were tested, and the results are expressed as mean hardness values (g).

### 4.5. Determination of Structural and Molecular Characteristics

#### 4.5.1. FTIR Spectroscopy

Bead samples were measured by FTIR spectroscopy using a Spectrum Two spectrometer (PerkinElmer, Bucks, UK), following the method of Ciarleglio et al. [50] with some modifications. Bead samples were dried at 40 °C until constant weight, ground into a fine powder using a mortar and pestle and mixed with potassium bromide (KBr) at a ratio of 1:100 (sample: KBr, *w*/*w*). The mixture was pressed into 10 mm pellets under vacuum using a hydraulic press. For each sample, 16 scans were conducted over the wavenumber range of 400–4000 cm^−1^, with a spectral resolution of 8 cm^−1^. The resulting spectrum was expressed in terms of transmittance (%).

#### 4.5.2. Morphological Observations

The surface morphological observations of the bead samples were carried out using a stereo microscope (Zeiss Stemi 508 with Axiocam 208 color camera, Carl Zeiss Microscopy GmbH, Göttingen, Germany) at magnifications of 0.63×, 2×, and 3.2×.

#### 4.5.3. Swelling Capacity

The swelling capacity of the bead samples was measured in accordance with the method of Gong et al. [87] and Kaprawet and Vatthanakul [85] with some modifications. Ten pre-dried beads were incubated at 37 °C for 3 h, then immersed in 50 mL of distilled water at 25 ± 1 °C under orbital agitation (100 rpm) for 30, 60, and 120 min. At each designated interval, beads were removed, gently blotted for 5 s with a clean paper towel to remove surface moisture, and immediately weighed using an electronic balance. The swelling capacity (%) at each time interval was calculated using the following formula:

(1) Swelling capacity (%)=(Wt−W0)/W0×100

#### 4.5.4. Encapsulation Efficiency

The encapsulation efficiency of the bead samples was tested using the method of Wongverawattanakul et al. [20]. For each treatment condition, the bead samples were prepared from 2 mL of the TPE-SA mixture that dissolved in 4 mL of sodium acetate solution (5% *w*/*v*). Then, the mixture was thoroughly stirred to facilitate a complete dissolution and followed by centrifugation for 5 min at 1000× *g*. The resulting supernatant was collected for the determination of total phenolic content (TPCb). Encapsulation efficiency (%) was calculated using the following equation:

(2) Encapsulation efficiency (%)=(TPCb/TPCe)×100

where TPCe was the total phenolic content of the TPE solution before the encapsulation, while TPCb was the total phenolic content in the disintegrated gel bead supernatant from the same concentration of the extract.

#### 4.5.5. ζ-Potential

ζ-potential was measured by following the method of using a Zetasizer Nano ZS (Malvern Instruments Ltd., Malvern, UK) equipped with a He–Ne laser (633 nm) and a detector angle of 173°. Beads were gently disintegrated and the dispersion diluted 1:20 in 1 mM potassium chloride. Electrophoretic mobility was determined by dynamic light scattering, and ζ-potential values were calculated and reported as mV as reported in Hunter [88].

### 4.6. Antioxidant Properties

#### 4.6.1. Total Phenolic Content (TPC)

The TPC of the bead mixture solution was determined using the Folin–Ciocalteu (FC) method following the procedure of Costanzo et al. [89]. A volume of 275 µL of the bead mixture was combined with 274 µL of FC reagent (1:10 dilution), followed by the addition of 1.450 mL of 700 mM sodium carbonate solution. The mixture was then incubated in the dark for 2 h, and the absorbance was measured at 765 nm using a UV–Vis spectrophotometer (Shimadzu F-15001, Kyoto, Japan). Results were calculated against a gallic acid standard curve and expressed as mg gallic acid equivalents per gram of dry weight (mg GAE/g DW).

#### 4.6.2. Total Flavonoid Content (TFC)

TFC level in the bead mixture was determined using aluminum chloride colorimetric assay by following the method of Costanzo et al. [89] with some modifications. In brief, 250 µL of bead mixture was combined with 75 µL of 5% sodium nitrate and incubated for 6 min at room temperature to initiate the reactions. After the incubation, 150 µL of 10% aluminum chloride, followed by 500 µL of 1 M NaOH, were added to the reaction mixture. Then, the final volume was adjusted to 1.525 mL with distilled water, and the absorbance was measured at 510 nm using a UV-Vis spectrophotometer (Shimadzu F-15001, Kyoto, Japan). Results were calculated against a quercetin standard curve and expressed as mg quercetin equivalents (QE) per gram of dry weight (mg QE/g DW).

#### 4.6.3. DPPH Radical Scavenging Activity

The DPPH free radical scavenging activity of the bead mixture was evaluated by following the method of Keawpeng et al. [90]. The reaction mixture consists of 0.1 mL of the bead mixture and 3.9 mL of a 60 μM DPPH solution. The reaction mixture was kept in the dark for 30 min, after which the absorbance was recorded at 515 nm using a UV–Vis spectrophotometer (UV-1800, Shimadzu Corporation, Kyoto, Japan). Antioxidant activity was expressed as the percentage inhibition of DPPH radicals.

#### 4.6.4. ABTS Radical Scavenging Activity

The ABTS radical scavenging activity in bead mixture samples was measured by following the method of Keawpeng et al. [90] with modifications. ABTS radical was generated by mixing 7 mM ABTS with 2.45 mM K_2_S_2_O_8_ and standing 12–16 h in the dark, then diluted to A_734_ = 0.70 ± 0.02. For analysis, the reaction mixture contains 1 mL of the bead mixture solution and 1 mL of ABTS reagent and is incubated in the dark at room temperature for 30 min. Absorbance was measured at 734 nm using a UV-visible spectrophotometer (Shimadzu UV-1800, Shimadzu Corporation, Kyoto, Japan). Results were expressed as the percentage of ABTS radical scavenging activity.

### 4.7. Antimicrobial Properties

Antimicrobial properties of the bead samples were tested with eight ATCC reference strains, including *Escherichia coli* ATCC 25922, *Staphylococcus aureus* ATCC 25923, *Listeria monocytogenes* ATCC 19115, *Salmonella enterica* ATCC 13076, *Pseudomonas aeruginosa* ATCC 27853, *Bacillus subtilis* ATCC 11774, *Candida albicans* ATCC 10231, and *Aspergillus niger* ATCC 16404. Strains were maintained as frozen glycerol stocks (15% *v*/*v*, −80 °C) and sub-cultured on Mueller–Hinton agar (MHA) for bacteria, Sabouraud Dextrose Agar (SDA) for yeast, and Potato Dextrose Agar (PDA) for fungi. Bacterial inocula were prepared according to Clinical and Laboratory Standards Institute (CLSI) guidelines [91] by adjusting 18–24 h colonies grown on MHA to a 0.5 McFarland standard (~1–2 × 10^8^ CFU/mL) and then diluting 1:20 in Mueller–Hinton broth (MHB) to obtain ~5 × 10^6^ CFU/mL. Yeast inocula (*C. albicans*) were prepared from 48 h SDA cultures and adjusted in sterile saline to 1–5 × 10^5^ CFU/mL, while *A. niger* spores were harvested from 7 d PDA cultures, filtered, and counted with a hemocytometer to ~1–5 × 10^6^ spores/mL. For contact assays, 0.5 g of SA–TPE beads was incubated with 10 mL of microbial suspension at 35 °C (bacteria) or 25 °C (fungi) under gentle agitation (100 rpm) for 24 h. Growth controls (inoculum without beads) and sterility controls (beads in saline) were included. At 0, 6, 12, and 24 h, 100 µL samples were withdrawn, neutralized in Dey/Engley broth, serially diluted (10^−1^ to 10^−6^), and spread-plated on MHA (bacteria) or SDA (fungi). Plates were incubated at 35 °C for 18–24 h (bacteria) or 25 °C for 48–72 h (fungi). Antimicrobial efficacy was expressed as log reduction.

### 4.8. Sensory Characterization

The sensory evaluation of the bead samples was performed using 50 untrained panelists aged between 20 and 60 years, using the 9-point hedonic scale ranging from 9 (like extremely) to 1 (dislike extremely), according to the method of Wichchukit and O’Mahony [92]. The samples tested were prepared with random three-digit codes and served to the panelists. The panelists were asked to evaluate the bead samples based on appearance, color, aroma, flavor, texture, and overall acceptability.

### 4.9. Statistical Analysis

All experiments were performed in triplicate unless otherwise stated. Exceptions include hardness analysis (20 individual beads per treatment), swelling capacity (10 beads per treatment), and sensory evaluation (50 untrained panelists). The results are expressed as mean ± standard deviation. A completely randomized design was applied to physicochemical, structural, molecular, and antioxidant property analyses, while a randomized complete block design was used for sensory evaluation to account for panelist variability. Statistical significance was determined using one-way analysis of variance, and mean separation was conducted with Duncan multiple range test at a significance level of *p* < 0.05. Data analysis was carried out using SPSS software (version 6, IBM Corp., Armonk, NY, USA).

## Data Availability

The original contributions presented in this study are included in the article. Further inquiries can be directed to the corresponding author.

## Abbreviations

a*	Red-green axis
ABTS	2,2′-azino-bis (3-ethylbenzthiazoline-6-sulphonic acid)
ATCC	The American Type Culture Collection
a_w_	Water activity
b*	Yellow-blue axis
BOD	Biochemical oxygen demand
CaCl_2_	Calcium chloride
COO^−^	Carboxylate
DPPH	2,2-diphenyl-1-picrylhydrazyl
DW	Dry weight
FC	Folin–Ciocalteu
FTIR	Fourier transform infrared spectroscopy
L*	Lightness
MHA	Mueller–Hinton agar
MHB	Mueller–Hinton broth
PDA	Potato dextrose agar
QE	Quercetin equivalent
SA	Sodium alginate
SA1	Sodium alginate at concentrations of 1%
SA2	Sodium alginate at concentrations of 2%
SA3	Sodium alginate at concentrations of 3%
SDA	Sabouraud dextrose agar
TFC	Total flavonoid content
TPC	Total phenolic content
TPCb	The total phenolic content in the disintegrated gel bead supernatant
TPCe	The total phenolic content of the TPE solution before the encapsulation
TPE	Tangerine peel extract
TPEen	TPE encapsulated
TPP	Tangerine peel powder
TSS	Total soluble solids
W0	The initial weight of the bead samples
Wt	The weight of the bead samples at each time interval
ζ	Zeta

## References

[1] Andrade A.M., Barbosa H.C., Shah A.M., Ahmad N., Vilarinho F., Khwaldia K., Silva S.A., Ramos F. (2022). Citrus by-products: Valuable source of bioactive compounds for food applications. Antioxidants.

[2] Negrea M., Cocan I., Jianu C., Alexa E., Berbecea A., Poiana M., Silivasan M. (2025). Valorization of citrus peel byproducts: A sustainable approach to nutrient-rich jam production. Foods.

[3] Zema A.D., Calabro S.P., Folino A., Tamburino V., Zappia G., Zimbone M.S. (2019). Wastewater management in citrus processing industries: An overview of advantages and limits. Water.

[4] Alam P., Alam A., Anwer M.K., Alqasoumi S.I. (2014). Quantitative estimation of hesperidin by HPTLC in different varieties of citrus peels. Asian Pac. J. Trop. Biomed..

[5] Martinidou E., Michailidis M., Ziogas V., Masuero D., Angeli A., Moysiadis T., Martens S., Ganopoulos I., Molassiotis A., Sarrou E. (2024). Comparative evaluation of secondary metabolite chemodiversity of citrus genebank collection in Greece: Can the peel be more than waste?. J. Agric. Food Chem..

[6] Magwaza L.S., Opara U.L., Cronje P.J., Landahl S., Ortiz J.O., Terry L.A. (2016). Rapid methods for extracting and quantifying phenolic compounds in citrus rinds. Food Sci. Nutr..

[7] Sung J., Suh H.J., Wamg Y. (2019). Effects of heat treatment of mandarin peel on flavonoid profiles and lipid accumulation in 3T3-L1 adipocytes. J. Food. Drug. Anal..

[8] Lin S., Simal-Gandara J., Cao H., Xiao J. (2023). The stability and degradation products of polyhydroxy flavonols in boiling water. Curr. Res. Food Sci..

[9] Saini K.R., Raamjit A., Sharma K., Prasad P., Shang X., Gowda M.G.K., Keum Y. (2022). Bioactive compounds of citrus fruits: A review of composition and health benefits of carotenoids, flavonoids, limonoids, and terpenes. Antioxidants.

[10] Wang Y., Guo Y., Zhang L., Yuan M., Zhao L., Bai C., McClements D.J. (2023). Impacts of hesperidin on whey protein functionality: Interacting mechanism, antioxidant capacity, and emulsion stabilizing effects. Front. Nutr..

[11] König A., Sadova N., Dornmayr M., Schwarzinger B., Neuhauser C., Stadlbauer V., Wallner M., Woischitzschläger J., Müller A., Tona R. (2023). Combined acid hydrolysis and fermentation improves bioactivity of citrus flavonoids in vitro and in vivo. Commun Biol..

[12] Rizki M.I., Agustina N.K.A., Sari A.K., Rahmatullah S.W., Isnani N. (2025). Characteristics and antioxidant activity of Limau kuit peel (*Citrus hystrix*) extract with variation of extraction solvent. Med. Lab. Technol. J..

[13] De Miera B.S., Cañadas R., González-Miquel M., González E.J. (2023). Recovery of phenolic compounds from orange peel waste by conventional and assisted extraction techniques using sustainable solvents. Front. Biosci..

[14] Rezagholizade-Shirvan A., Soltani M., Shokri S., Radfar R., Arab M., Shamloo E. (2024). Bioactive compound encapsulation: Characteristics, applications in food systems, and implications for human health. Food Chem. X.

[15] Pérez-Pérez V., Jiménez-Martínez C., González-Escobar J.L., Corzo-Ríos L.J. (2024). Exploring the impact of encapsulation on the stability and bioactivity of peptides extracted from botanical sources: Trends and opportunities. Front. Chem..

[16] Abka-Khajouei R., Tounsi L., Shahabi N., Patel A.K., Abdelkafi S., Michaud P. (2022). Structures, properties and applications of alginates. Mar. Drugs.

[17] Kavand A., Noverraz F., Gerber-Lemaire S. (2024). Recent advances in alginate-based hydrogels for cell transplantation applications. Pharmaceutics.

[18] Milivojević M., Popović A., Pajić-Lijaković I., Šoštarić I., Kolašinac S., Stevanović Z.D. (2023). Alginate gel-based carriers for encapsulation of carotenoids: On challenges and applications. Gels.

[19] Dhamecha D., Movsas R., Sano U., Menon J.U. (2019). Applications of alginate microspheres in therapeutics delivery and cell culture: Past, present and future. Int. J. Pharm..

[20] Wongverawattanakul C., Suklaew P.O., Chusak C., Adisakwattana S., Thilavech T. (2022). Encapsulation of *Mesona chinensis* benth extract in alginate beads enhances the stability and antioxidant activity of polyphenols under simulated gastrointestinal digestion. Foods.

[21] Qiu K., Huang Y., Anselmo A.C. (2021). Polymer and crosslinker content influences performance of encapsulated live biotherapeutic products. Cell. Mol. Bioeng..

[22] Abbasiliasi S., Shun T.J., Ibrahim T.A.T., Ismail N., Ariff A.B., Mokhtar N.K., Mustafa S. (2019). Use of sodium alginate in the preparation of gelatin-based hard capsule shells and their evaluation in vitro. RSC Adv..

[23] Colin C., Akpo E., Perrin A., Cornu D., Cambedouzou J. (2024). Encapsulation in alginates hydrogels and controlled release: An overview. Molecules.

[24] Lai J., Azad A.K., Sulaiman W.M.A.W., Kumarasamy V., Subramaniyan V., Alshehade S.A. (2024). Alginate-based encapsulation fabrication technique for drug delivery: An updated review of particle type, formulation technique, pharmaceutical ingredient, and targeted delivery system. Pharmaceutics.

[25] Ma W.-L., Mou C.-L., Chen S.-H., Li Y.-D., Deng H.-B. (2022). A mild method for encapsulation of citral in monodispersed alginate microcapsules. Polymers.

[26] Dhasmana A., Preetam S., Malik S., Jadon V.S., Joshi N., Bhandari G., Gupta S., Mishra R., Rustagi S., Samal S.K. (2024). Revitalizing elixir with orange peel amplification of alginate fish oil beads for enhanced anti-aging efficacy. Sci. Rep..

[27] Julaeha E., Puspita W.R., Permadi N., Harja A., Nurjanah S., Wahyudi T., Al-Anshori J. (2024). Optimization of *Citrus aurantifolia* peel extract encapsulation in alginate-gelatin hydrogel microbeads for antibacterial wound dressing applications. Carbohydr. Polym. Technol. Appl..

[28] Mostaghimi M., Majdinasab M., Golmakani M.T., Hadian M., Hosseini S.M.H. (2023). Development and characterization of antimicrobial alginate hydrogel beads filled with cinnamon essential oil nanoemulsion. J. Biomater. Sci. Polym. Ed..

[29] Kaur N., Singh B., Sharma S. (2018). Hydrogels for potential food application: Effect of sodium alginate and calcium chloride on physical and morphological properties. Pharm. Innov. J..

[30] Xiao Y., Wang L., Zhang X., Ren Y., Wang J., Niu B., Li W. (2024). Preparation and characterization of silica-coated sodium alginate hydrogel beads and the delivery of curcumin. J. Biomater. Sci. Polym. Ed..

[31] Kowalski G., Witczak M., Kuterasiński Ł. (2024). Structure effects on swelling properties of hydrogels based on sodium alginate and acrylic polymers. Molecules.

[32] Stachowiak N., Kowalonek J., Kozlowska J., Burkowska-But A. (2023). Stability studies, biodegradation tests, and mechanical properties of sodium alginate and gellan gum beads containing surfactant. Polymers.

[33] Chuang J.J., Huang Y.Y., Lo S.H., Hsu T.F., Huang W.Y., Huang S.L., Lin Y.S. (2017). Effects of pH on the shape of alginate particles and its release behavior. Int. J. Polym. Sci..

[34] Malektaj H., Drozdov A.D., de Claville Christiansen J. (2023). Swelling of homogeneous alginate gels with multi-stimuli sensitivity. Int. J. Mol. Sci..

[35] Makarova A.O., Derkach S.R., Khair T., Kazantseva M.A., Zuev Y.F., Zueva O.S. (2023). Ion-induced polysaccharide gelation: Peculiarities of alginate egg-box association with different divalent cations. Polymers.

[36] Łętocha A., Miastkowska M., Sikora E. (2022). Preparation and characteristics of alginate microparticles for food, pharmaceutical and cosmetic applications. Polymers.

[37] Agles A.A., Bourg I.C. (2024). Structure and dynamics of water in polysaccharide (alginate) solutions and gels explained by the core–shell model. Biomacromolecules.

[38] Ji D., Park J.M., Oh M.S., Nguyen T.L., Shin H., Kim J.S., Kim D., Park H.S., Kim J. (2022). Superstrong, superstiff, and conductive alginate hydrogels. Nat. Commun..

[39] Savic I.M., Savic Gajic I.M., Milovanovic M.G., Zerajic S., Gajic D.G. (2022). Optimization of ultrasound-assisted extraction and encapsulation of antioxidants from orange peels in alginate-chitosan microparticles. Antioxidants.

[40] Tomić S.L., Babić Radić M.M., Vuković J.S., Filipović V.V., Nikodinovic-Runic J., Vukomanović M. (2023). Alginate-based hydrogels and scaffolds for biomedical applications. Mar. Drugs.

[41] Bennacef C., Desobry S., Jasniewski J., Leclerc S., Probst L., Desobry-Banon S. (2023). Influence of alginate properties and calcium chloride concentration on alginate bead reticulation and size: A phenomenological approach. Polymers.

[42] Moya M.L., Morley M., Khanna O., Opara E.C., Brey E.M. (2012). Stability of alginate microbead properties in vitro. J. Mater. Sci. Mater. Med..

[43] Aldawsari M.F., Ahmed M.M., Fatima F., Anwer M.K., Katakam P., Khan A. (2021). Development and characterization of calcium-alginate beads of apigenin: In vitro antitumor, antibacterial, and antioxidant activities. Mar. Drugs.

[44] Frent O.D., Vicas L.G., Duteanu N., Morgovan C.M., Jurca T., Pallag A., Muresan M.E., Filip S.M., Lucaciu R.L., Marian E. (2022). Sodium alginate—Natural microencapsulation material of polymeric microparticles. Int. J. Mol. Sci..

[45] Zhang C., Grossier R., Candoni N., Veesler S. (2021). Preparation of alginate hydrogel microparticles by gelation introducing cross-linkers using droplet-based microfluidics: A review of methods. Biomater. Res..

[46] Grassi M., Sandolo C., Perin D., Coviello T., Lapasin R., Grassi G. (2009). Structural characterization of calcium alginate matrices by means of mechanical and release tests. Molecules.

[47] Jeong C., Kim S., Lee C., Cho S., Kim S.B. (2020). Changes in the physical properties of calcium alginate gel beads under a wide range of gelation temperature conditions. Foods.

[48] Belattmania Z., Kaidi S., El Atouani S., Katif C., Bentiss F., Jama C., Reani A., Sabour B., Vasconcelos V. (2020). Isolation and FTIR-ATR and 1H NMR characterization of alginates from the main alginophyte species of the atlantic coast of Morocco. Molecules.

[49] Helmiyati, Aprilliza M. (2017). Characterization and properties of sodium alginate from brown algae used as an ecofriendly superabsorbent. IOP Conf. Ser. Mater. Sci. Eng..

[50] Ciarleglio G., Cinti F., Toto E., Santonicola M.G. (2023). Synthesis and characterization of alginate gel beads with embedded zeolite structures as carriers of hydrophobic curcumin. Gels.

[51] Khorshidian N., Mahboubi A., Kalantari N., Hosseini H., Yousefi M., Arab M., de Cruz A.G., Mortazavian A.M., Mahdavi F.S. (2019). Chitosan-coated alginate microcapsules loaded with herbal galactagogue extract: Formulation optimization and characterization. Iran J. Pharm. Res..

[52] Stanisławska N., Khachatryan G., Khachatryan K., Krystyjan M., Makarewicz M., Krzan M. (2023). Formation and investigation of physicochemical and microbiological properties of biocomposite films containing turmeric extract nano/microcapsules. Polymers.

[53] Galogahi F.M., Tran D.T., Ouyang L., Nguyen N.T. (2025). Generation of oil-in-water emulsion-core sodium alginate beads. Macromol. Chem. Phys..

[54] Prasetyaningrum A., Rokhati N., Djaeni M., Kumoro A.C., Purwati D., Hakiim A., Ashianti A.D., Utomo D.P. (2024). Effect of cross-linking agents on sodium alginate-based quercetin beads: Physicochemical properties and controlled release kinetics. Food Res..

[55] Schuster E., Eckardt J., Hermansson A.M., Larsson A., Lorén N., Altskär A., Ström A. (2014). Microstructural, mechanical and mass transport properties of isotropic and capillary alginate gels. Soft Matter.

[56] Besiri I.N., Goudoulas T.B., Fattahi E., Becker T. (2023). Experimental advances in the real-time recording of cross-linking alginate in situ gelation: A Review. Polymers.

[57] Savić Gajić I.M., Savić I.M., Svirčev Z. (2023). Preparation and characterization of alginate hydrogels with high water-retaining capacity. Polymers.

[58] Zhan L., Lin Z., Li W., Qin Y., Sun Q., Ji N., Xie F. (2024). The construction of sodium alginate/carboxymethyl chitosan microcapsules as the physical barrier to reduce corn starch digestion. Foods.

[59] Sankalia M.G., Mashru R.C., Sankalia J.M., Sutariya V.B. (2005). Papain entrapment in alginate beads for stability improvement and site-specific delivery: Physicochemical characterization and factorial optimization using neural network modeling. AAPS Pharm. Sci. Tech..

[60] Essifi K., Brahmi M., Berraaouan D., Ed-Daoui A., El Bachiri A., Fauconnier M.L., Tahani A. (2021). Influence of sodium alginate concentration on microcapsules properties foreseeing the protection and controlled release of bioactive substances. J. Chem..

[61] Midekessa G., Godakumara K., Ord J., Viil J., Lattekivi F., Dissanayake K., Kopanchuk S., Rinken A., Andronowska A., Bhattacharjee S. (2020). Zeta potential of extracellular vesicles: Toward understanding the attributes that determine colloidal stability. ACS Omega.

[62] Aziz S.N., Badawy A.A., Nessem D.I., Abd El Malak N.S., Naguib M.J. (2023). Chitosan-coated alginate (CCA) nanoparticles for augmentation of topical antihistaminic activity of diphenhydramine: In-vitro optimization, skin histopathology and pharmacodynamic studies with in vitro/in vivo correlation. Drug. Dev. Ind. Pharm..

[63] Sepúlveda-Rivas S., Fritz H.F., Valenzuela C., Santiviago C.A., Morales J.O. (2019). Development of novel EE/alginate polyelectrolyte complex nanoparticles for lysozyme delivery: Physicochemical properties and in vitro safety. Pharmaceutics.

[64] Amin M.K., Boateng J.S. (2022). Enhancing stability and mucoadhesive properties of chitosan nanoparticles by surface modification with sodium alginate and polyethylene glycol for potential oral mucosa vaccine delivery. Mar. Drugs.

[65] Ibrahim H.M., Awad M., Al-Farraj A.S., Al-Turki A.M. (2020). Stability and dynamic aggregation of bare and stabilized zero-valent iron nanoparticles under variable solution chemistry. Nanomaterials.

[66] Chen G.L., Cai H.Y., Chen J.P., Li R., Zhong S.Y., Jia X.J., Liu X.F., Song B.B. (2022). Chitosan/alginate nanoparticles for the enhanced oral antithrombotic activity of clam heparinoid from the clam *Coelomactra antiquata*. Mar. Drugs.

[67] Hassani A., Mahmood S., Enezei H.H., Hussain S.A., Hamad H.A., Aldoghachi A.F., Hagar A., Doolaanea A.A., Ibrahim W.N. (2020). Formulation, characterization and biological activity screening of sodium alginate-gum arabic nanoparticles loaded with curcumin. Molecules.

[68] Wang H., Wang P., Wang F., Chen H., Chen L., Hu Y., Liu Y. (2024). Integrated HS-GC–IMS and UPLC-Q-Orbitrap HRMS-based metabolomics revealed the characteristics and differential volatile and nonvolatile metabolites of different citrus peels. Curr. Res. Food Sci..

[69] Feng Q., Fan B., He Y.C. (2024). Antibacterial, antioxidant, Cr (VI) adsorption and dye adsorption effects of biochar-based silver nanoparticles-sodium alginate-tannic acid composite gel beads. Int. J. Biol. Macromol..

[70] Jing Q., Ma Y., He J., Ren Z. (2023). Highly stable, mechanically enhanced, and easy-to-collect Sodium alginate/NZVI-rGO gel beads for efficient removal of Cr (VI). Polymers.

[71] Martinović J., Lukinac J., Jukić M., Ambrus R., Planinić M., Šelo G., Klarić A.M., Perković G., Bucić-Kojić A. (2023). Physicochemical characterization and evaluation of gastrointestinal in vitro behavior of alginate-based microbeads with encapsulated grape pomace extracts. Pharmaceutics.

[72] Flamminii F., Paciulli M., Di Michele A., Littardi P., Carini E., Chiavaro E., Pittia P., Di Mattia C.D. (2021). Alginate-based microparticles structured with different biopolymers and enriched with a phenolic-rich olive leaves extract: A physico-chemical characterization. Curr. Res. Food Sci..

[73] Sabry B.A., Badr A.N., Mohammed D.M., Desoukey M.A., Farouk A. (2024). Validating the protective role of orange and tangerine peel extracts foramending food safety against microorganisms’ contamination using molecular docking. Heliyon.

[74] Shehata M.G., Awad T.S., Asker D., El Sohaimy S.A., Abd El-Aziz N.M., Youssef M.M. (2021). Antioxidant and antimicrobial activities and UPLC-ESI-MS/MS polyphenolic profile of sweet orange peel extracts. Curr. Res. Food Sci..

[75] Al hamza Hasoon B.A., Mahmood B.S., Mohamed E.A., Jabir M.S., Jawad K.H., Hussein N.N., Sulaiman G.M., Dewir Y.H., Mendler-Drienyovszki N. (2024). Tangerine fruit peel extract mediated biogenic synthesized silver nanoparticles and their potential antimicrobial, antioxidant, and cytotoxic assessments. Green Process. Synth..

[76] Rijo P., Matias D., Fernandes A.S., Simões M.F., Nicolai M., Reis C.P. (2014). Antimicrobial plant extracts encapsulated into polymeric beads for potential application on the skin. Polymers.

[77] Tøndervik A., Sletta H., Klinkenberg G., Emanuel C., Powell L.C., Pritchard M.F., Khan S., Craine K.M., Onsøyen E., Rye P.D. (2014). Alginate oligosaccharides inhibit fungal cell growth and potentiate the activity of antifungals against *Candida* and *Aspergillus* spp.. PLoS ONE.

[78] Aguirre-Calvo T.R., Sosa N., López T.A., Quintanilla-Carvajal M.X., Perullini M., Santagapita P.R. (2022). Bioaccessibility assay, antioxidant activity and consumer-oriented sensory analysis of Beta vulgaris by-product encapsulated in Ca (II)-alginate beads for different foods. Food Chem. Mol. Sci..

[79] Sobri A.M., Seow L.J., Issara U., Ab Rahman N.A., Huda N., Seow E.K. (2023). Effect of sodium alginate on the physicochemical and sensory properties of vegan surimi. Canrea J. Food Technol. Nutr. Culin. J..

[80] Stribiţcaia E., Krop E.M., Lewin R., Holmes M., Sarkar A. (2020). Tribology and rheology of bead-layered hydrogels: Influence of bead size on sensory perception. Food Hydrocoll..

[81] Farahani Z.K., Mousavi M., Ardebili S.M.S., Bakhoda H. (2022). Modification of sodium alginate by octenyl succinic anhydride to fabricate beads for encapsulating jujube extract. Curr. Res. Food Sci..

[82] Uzokboev S., Akhmadbekov K., Nuritdinova R.N., Tawfik S.M., Lee Y.I. (2024). Unveiling the potential of alginate-based nanomaterials in sensing technology and smart delivery applications. Beilstein J. Nanotechnol..

[83] Sunarharum W.B., Kambodji A.D., Nur M. (2020). The physical properties of coffee caviar as influenced by sodium alginate concentration and calcium sources. IOP Conf. Ser. Earth Environ. Sci..

[84] Sharma P., Dadwal V., Rahmatkar S.N., Gupta M., Singh D. (2022). Flavonoid composition and antioxidant efficacy of citrus peels: An integrated in vitro and in Silico approach toward potential neuroprotective agents. J. Sci. Ind. Res..

[85] Kamprawet P., Vatthanakul S. (2018). Producing passion fruit beads by reverse spherification technique. Thai Sci. Technol. J..

[86] Phawaphuthanon N., Behnam S., Koo S.Y., Pan C.H., Chung D. (2014). Characterization of core–shell calcium-alginate macrocapsules fabricated by electro-coextrusion. Int. J. Biol. Macromol..

[87] Gong R., Li C., Zhu S., Zhang Y., Du Y., Jiang J. (2011). A novel pH-sensitive hydrogel based on dual crosslinked alginate/N-α-glutaric acid chitosan for oral delivery of protein. Carbohydr. Polym..

[88] Hunter R.J. (2013). Zeta Potential in Colloid Science: Principles and Applications.

[89] Costanzo G., Vitale E., Iesce M., Naviglio D., Amoresano A., Fontanarosa C., Spinelli M., Ciaravolo M., Arena C. (2022). Antioxidant properties of pulp, peel and seeds of Phlegrean mandarin (*Citrus reticulata* Blanco) at different stages of fruit ripening. Antioxidants.

[90] Keawpeng I., Paulraj B., Venkatachalam K. (2022). Antioxidant and antimicrobial properties of mung bean phyto-film combined with longkong pericarp extract and sonication. Membranes.

[91] Clinical and Laboratory Standards Institute (2018). Methods for Dilution Antimicrobial Susceptibility Tests for Bacteria that Grow Aerobically.

[92] Wichchukit S., O’Mahony M. (2015). The 9-point hedonic scale and hedonic ranking in food science: Some reappraisals and alternatives. J. Sci. Food Agric..

